# Survival and causes of death in patients with alpha and beta-thalassemia in Northern Thailand

**DOI:** 10.1080/07853890.2024.2338246

**Published:** 2024-04-11

**Authors:** Adisak Tantiworawit, Thansita Kamolsripat, Pokpong Piriyakhuntorn, Thanawat Rattanathammethee, Sasinee Hantrakool, Chatree Chai-Adisaksopha, Ekarat Rattarittamrong, Lalita Norasetthada, Kanda Fanhchaksai, Pimlak Charoenkwan

**Affiliations:** a Division of Hematology, Department of Internal Medicine, Faculty of Medicine, Chiang Mai University, Chiang Mai, Thailand; b Thalassemia and Hematology Center, Faculty of Medicine, Chiang Mai University, Chiang Mai, Thailand; c Division of Hematology and Oncology, Department of Pediatrics, Faculty of Medicine, Chiang Mai University, Chiang Mai, Thailand

**Keywords:** Cardiac complications, infection, iron overload, mortality, thalassemia, survival

## Abstract

**Background:**

Thalassemia is the most prevalent hereditary anaemia worldwide. Severe forms of thalassemia can lead to reduced life expectancy due to disease-related complications.

**Objectives:**

To investigate the survival of thalassemia patients across varying disease severity, causes of death and related clinical factors.

**Patients and methods:**

We conducted a retrospective review of thalassemia patients who received medical care at Chiang Mai University Hospital. The analysis focused on survival outcomes, and potential associations between clinical factors and patient survival.

**Results:**

A total of 789 patients were included in our study cohort. Among them, 38.1% had Hb H disease, 35.4% had Hb E/beta-thalassemia and 26.5% had beta-thalassemia major. Half of the patients (50.1%) required regular transfusions. Sixty-five patients (8.2%) had deceased. The predominant causes of mortality were infection-related (36.9%) and cardiac complications (27.7%). Transfusion-dependent thalassemia (TDT) (adjusted HR 3.68, 95% CI 1.39–9.72, *p =* 0.008) and a mean serum ferritin level ≥3000 ng/mL (adjusted HR 4.18, 95% CI 2.20–7.92, *p* < 0.001) were independently associated with poorer survival.

**Conclusions:**

Our study highlights the primary contributors to mortality in patients with thalassemia as infection-related issues and cardiac complications. It also underscores the significant impact of TDT and elevated serum ferritin levels on the survival of thalassemia patients.

## Background

Thalassemia is the most common hereditary anaemia worldwide. The prevalence is high in the malaria-endemic areas, including Southeast Asia, and Thailand. The condition is caused by mutations on globin genes, resulting in decreased or absent production of globin protein, thereby leading to ineffective erythropoiesis and varying degrees of chronic haemolytic anaemia [1]. Patients with severe thalassemia require long-term regular red blood cell transfusions and are classified as having transfusion-dependent thalassemia (TDT). Patients with TDT need iron chelation for transfusional iron overload, and monitoring of complications. Common complications in patients with TDT are related to iron overload, of which the major ones are cardiomyopathy, liver cirrhosis and hormonal deficiencies [1,2]. On the other hand, patients with thalassemia of mild to moderate severity, who do not require long-term transfusions, are classified as having non-transfusion-dependent thalassemia (NTDT). Complications typically observed in patients with NTDT are related to chronic haemolysis and include conditions such as pulmonary artery hypertension, extramedullary haematopoiesis, cholelithiasis and iron overload resulting from increased gastrointestinal iron absorption [1,3]. Furthermore, patients with thalassemia are also at higher risk of infections due to the immunological disturbances related to both the disease itself and its treatments [4–6].

Over the past three decades, the survival rates of patients with thalassemia, particularly those with TDT, have exhibited significant improvements owing to regular red blood cell transfusions and the use of iron chelators [7–20]. The most distinctive change has been the reduction in the iron-overloaded cardiomyopathy and cardiac failure as leading causes of mortality. This improvement can be attributed to the availability of iron chelation therapy and the implementation of MRI-based techniques for monitoring tissue iron overload [18,19]. Iron overload remains the major factor related to mortality in patients with TDT, and the current effective management of iron overload has resulted in more favourable outcomes. Additionally, other significant contributors to mortality in patients with TDT include infections, liver diseases, diabetes mellitus and thromboembolisms [9,13]. For patients with NTDT, a recent study of a large cohort of non-transfusion-dependent beta-thalassemia patients revealed that cardiovascular disease was the major cause of early death, whereas hepatic disease was the major cause of death in older patients [21].

In northern Thailand, the main types of thalassemia diseases are haemoglobin (Hb) E/beta-thalassemia, beta-thalassemia major and Hb H disease [22]. In this study, our aim was to investigate the survival and causes of mortality among patients with thalassemia, across a spectrum of disease severity from NTDT to TDT. We also sought to explore the factors that influence survival. The information will be useful for improving the treatment outcomes for patients with thalassemia.

## Patients and methods

This study adhered to the guidelines set forth in the 1975 Declaration of Helsinki, which outlines ethical principles for medical research involving human subjects, and received approval from the local Institutional Research Ethics Committee at the Faculty of Medicine, Chiang Mai University (Study code No. MED-2561-05762 and MED-2566-09438). Informed consent was waived as the study involved no more than minimal risk.

A retrospective review of patients diagnosed with thalassemia was conducted using the divisional registries of the Division of Hematology within the Department of Internal Medicine and the Department of Pediatrics at Chiang Mai University Hospital. The inclusion criteria comprised patients diagnosed with Hb H disease, beta-thalassemia major and Hb E/beta-thalassemia. Both deletional and non-deletional forms of Hb H disease were classified as Hb H disease. Additionally double heterozygosity of Hb H disease with heterozygous beta-globin gene mutation such as AEBart’s disease and AEBart’s Constant Spring disease, were also categorized under Hb H disease. Double heterozygosity of Hb E/beta-thalassemia and Hb H disease (EFBart’s disease) was classified within the Hb E/beta-thalassemia group. Exclusion criteria comprised other types of thalassemia and Hb variants, including homozygous Hb Constant Spring, and Hb variant/beta-thalassemia, as well as cases with incomplete medical records where information regarding survival status and cause of death was unavailable.

Thalassemia diagnosis was confirmed by reviewing the results of Hb analysis and/or globin gene analyses. The medical records of the patients were retrospectively reviewed. The data collection included the following: survival status, age, sex, type of thalassemia, transfusion dependency (NTDT defined as less than an average of three transfusions per year [23]), iron chelation status, mean pre-transfusion Hb level and mean serum ferritin level (calculated from the last five consecutive measurements), splenectomy status and the presence of coexisting diseases. These coexisting diseases comprised viral hepatitis B and C infections, reduced left ventricular ejection fraction (LVEF) defined as LVEF less than 40% by echocardiography [24], pulmonary hypertension as defined by a peak tricuspid regurgitation velocity exceeding 3.4 m/s by echocardiography [25], liver cirrhosis as diagnosed by abdominal ultrasonography, computerized tomography or magnetic resonance imaging, diabetes mellitus, hypothyroidism, hypoparathyroidism, hypogonadism, and extramedullary haematopoiesis confirmed by laboratory or imaging criteria or documented in the medical records.

Survival status was determined by referencing medical records and cross-referenced with the Thai National database (Official statistics registration system). In cases where patients had died, data regarding the causes of death was extracted from both medical records and the Thai National database. Survival analysis was conducted. Clinical factors were analyzed for potential association with the survival.

The frequencies were reported as number and percentage. The Pearson’s Chi-square test or Fisher’s Exact test was used to compare the parameters between the alive and deceased patient groups. Survival analysis was conducted using the Kaplan–Meier method, defining the event as death from any cause, with time to event calculated as the duration from birth to death. For patients who survived, the follow-up period concluded at the time of data collection. The log-rank test was used to compare survival time among the patient groups. Cox regression analysis was used to determine the significant factors associated with survival. A statistically significant difference was defined at a *p* value <0.05.

In the initial univariable Cox regression analysis, characteristics with a *p* value of <0.1 were considered for inclusion in a forward stepwise model selection process, ultimately leading to a final multivariable Cox regression analysis. All statistical analyses were carried out using IBM SPSS Statistics for Windows, Version 22.0 (Armonk, NY: IBM Corp., USA).

## Results

We examined a cohort of 789 patients with thalassemia from the divisional registries of the Division of Hematology within the Department of Internal Medicine (February 1979–September 2022) and the Department of Pediatrics (January 2002–September 2022) at Chiang Mai University Hospital. Among these patients, 400 (50.7%) were male, and the median age (IQR) at the end of follow-up was 18.8 (11.7) years. The demographic and clinical characteristics of the study patients at the end of the follow-up period are shown in Table 1. Within the cohort, 301 patients (38.1%) were diagnosed with Hb H disease, 279 patients with Hb E/beta-thalassemia (35.4%) and 209 patients with beta-thalassemia major (26.5%). Half of the patients (395, 50.1%) were classified as having TDT.

**Table 1.** Demographic and clinical characteristics of the study patients.

Patient characteristics	Total
	(*n* = 789)
*Gender, n (%)*	
Male	400 (50.7)
Female	389 (49.3)
*Thalassemia type, n (%)*	
Beta-thalassemia major	209 (26.5)
Hb E/beta-thalassemia	279 (35.4)
Hb H disease	301 (38.1)
*Transfusion dependency, n (%)*	
Dependent	395 (50.1)
Non-dependent	394 (49.9)
*Splenectomy, n (%)*	
Yes	233 (29.5)
No	556 (70.5)
*Iron chelation, n (%)*	
Yes	336 (42.6)
No	453 (57.4)
*Steady-state or pre-transfusion Hb, n (%)*	
<7 g/dL	132 (16.7)
≥7 g/dL	657 (83.3)
*Mean serum ferritin level, n (%)*	
≥3000 ng/mL	84 (16.4)
<3000 ng/mL	427 (83.6)
Year of birth median (IQR)	2002 (13)
Follow-up duration in the hospital, years median (IQR)	13.2 (10.6)
Age at the end of follow-up, years median (IQR)	18.8 (11.7)

Sixty-five patients (8.2%) in the cohort had deceased, with a mean age at death of 17.0 ± 0.9 years. Table 2 shows the causes of death in 65 patients. The major causes of death were the infection-related conditions (24, 36.9%), followed by cardiac complications (18, 27.7%), thalassemia/anaemia (9, 13.8%), trauma (4, 6.2%) and other causes (10, 15.4%).

**Table 2.** Causes of death as classified by the thalassemia disease type.

Causes	Number (%)	Beta-thalassemia major	Hb E/beta-thalassemia	Hb H disease
Infections-related	24 (36.9)	13 (36.1)	9 (47.4)	2 (20.0)
Cardiac complications	18 (27.7)	11 (30.6)	3 (15.8)	4 (40.0)
Heart failure and dilated cardiomyopathy	15	10	1	4
Coronary artery diseases	2	1	1	0
Severe pulmonary hypertension	1	0	1	0
Thalassemia/anaemia	9 (13.8)	6 (16.6)	3 (15.8)	0
Trauma	4 (6.2)	2 (5.6)	1 (5.2)	1 (10.0)
Others	10 (15.4)	4 (11.1)	3 (15.8)	3 (30.0)
Thrombotic-related complications	2	0	1	1
Diabetes mellitus/diabetic ketoacidosis	2	2	0	0
Relapse ALL	1	1	0	0
Systemic lupus erythematosus	1	0	0	1
Rheumatoid arthritis	1	0	1	0
Hypotension	1	0	1	0
Asthma	1	1	0	0
Kidney failure	1	0	0	1
Total	65 (100)	36 (100)	19 (100)	10 (100)

Table 3 shows the comparison of clinical parameters and complications based on the patients’ survival status. There were significant differences between the groups of patients who were alive and those who had deceased, concerning the type of thalassemia, transfusion dependency status, pre-transfusion haemoglobin levels (<7 g/dL or >7 g/dL), mean serum ferritin levels (≥3000 ng/mL or <3000 ng/mL) and the presence of complications, including reduced LVEF <40%, pulmonary hypertension, diabetes mellitus and extramedullary haematopoiesis.

**Table 3.** Comparison of clinical parameters and complications according to survival status.

Patient characteristics	Alive	Deceased	*p* Value
	(*n* = 724)	(*n* = 65)	
*Gender, n (%)*			
Male	368 (50.8)	32 (49.2)	0.805
Female	356 (49.2)	33 (50.8)	
*Thalassemia type, n (%)*			
Beta-thalassemia major	173 (23.9)	36 (55.4)	<0.001*
Hb E/beta-thalassemia	260 (35.9)	19 (29.2)	
Hb H disease	291 (40.2)	10 (15.4)	
*Transfusion dependency, n (%)*			
Dependent	344 (47.5)	51 (78.5)	<0.001*
Non-dependent	380 (52.5)	14 (21.5)	
*Iron chelation, n (%)*			
Yes	303 (41.9)	33 (50.8)	0.164
No	421 (58.1)	32 (49.2)	
*Steady-state or pre-transfusion Hb, n (%)*			
<7 g/dL	106 (14.6)	26 (40.0)	<0.001*
≥7 g/dL	618 (85.4)	39 (60.0)	
*Mean serum ferritin level, n (%)*			
≥3000 ng/mL	62 (13.2)	22 (52.4)	<0.001*
<3000 ng/mL	407 (86.8)	20 (47.6)	
*Complications, n (%)*			
Hepatitis B infection	9 (1.2)	1 (1.5)	0.593
Hepatitis C infection	10 (1.3)	1 (1.5)	0.956
Reduced LVEF <40%	15 (2.0)	12 (18.4)	0.003*
Pulmonary hypertension	23 (3.1)	11 (16.9)	0.042*
Liver cirrhosis	1 (0.1)	1 (1.5)	0.161
Diabetes mellitus	13 (1.8)	5 (7.7)	0.002*
Hypothyroidism	24 (3.3)	4 (6.1)	0.268
Hypoparathyroidism	7 (0.9)	2 (3.0)	0.174
Hypogonadism	22 (3.0)	4 (6.1)	0.071*
Adrenal insufficiency	43 (5.9)	2 (3.0)	0.571
Extramedullary haematopoiesis	23 (3.1)	1 (1.5)	<0.001*
Venous thrombosis	4 (0.6)	0	0.709

LVEF, left ventricular ejection fraction.

* Statistically significant results.

**Figure 1.**
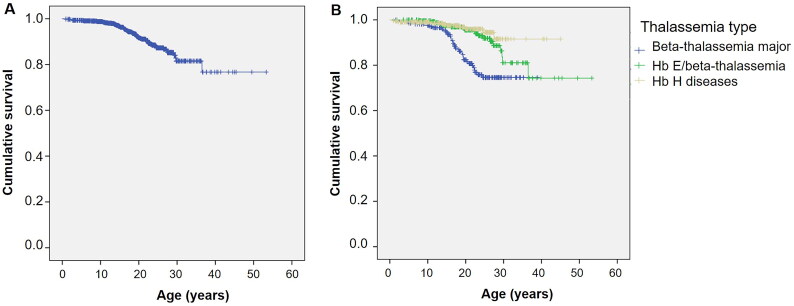
Kaplan–Meier survival curve of thalassemia patients. (A) Kaplan–Meier survival curve of all thalassemia patients. (B) Kaplan–Meier survival curve of thalassemia patients as classified by thalassemia type.

**Figure 2.**
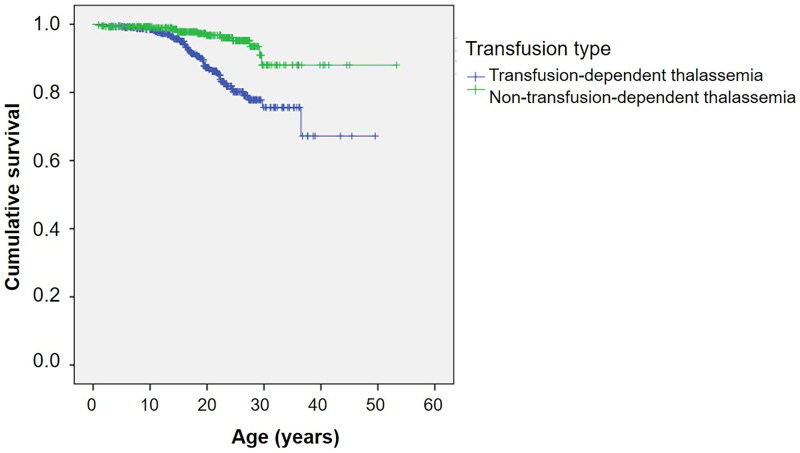
Kaplan–Meier survival curve of thalassemia patients as classified by transfusion status.

**Figure 3.**
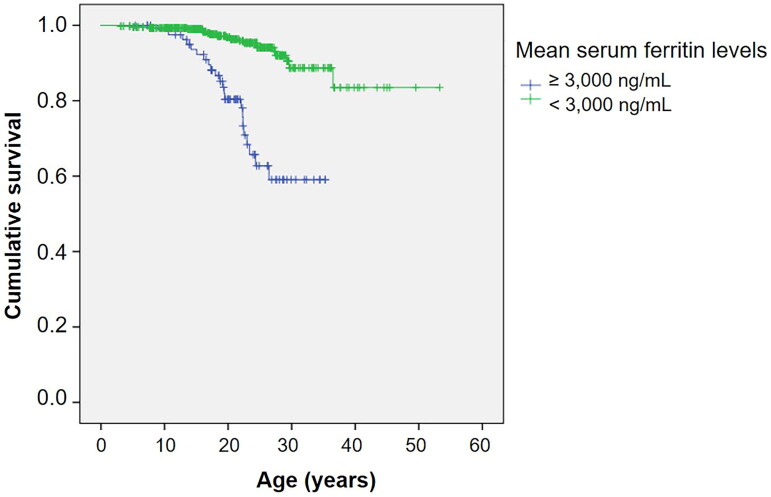
Kaplan–Meier survival curve of thalassemia patients as classified by mean serum ferritin levels.

Figure 1A shows the Kaplan–Meier survival curve for all thalassemia patients. The estimated median survival was not reached for the overall group. Figure 1B shows survival curves stratified by the type of thalassemia. Survival curves according to the transfusion status and mean serum ferritin levels are shown in Figures 2 and 3, respectively.

The univariable and multivariable Cox regression model results, assessing significant factors influencing survival in thalassemia patients, are summarized in Tables 4 and 5, respectively. TDT and mean serum ferritin levels ≥3000 ng/mL were independently associated with poorer survival.

**Table 4.** Univariable cox regression model results of significant factors (*p* value <0.1) affecting survivals in thalassemia patients.

Variables	Hazard ratio (95% CI)	*p* Value
Thalassemia type (beta-thalassemia major vs. Hb H disease)	4.15 (2.06–8.38)	<0.001*
(Hb E/beta-thalassemia vs. Hb H disease)	1.56 (0.72–3.37)	0.251
Transfusion type (TDT vs. NTDT)	3.45 (1.91–6.23)	<0.001*
Hemoglobin level (<7 g/dL vs. ≥7 g/dL)	2.16 (1.31–3.56)	0.002*
Mean serum ferritin level (≥ 3000 ng/mL vs. <3000 ng/mL)	5.93 (3.20–10.99)	<0.001*

NTDT, non-transfusion-dependent thalassemia; TDT, transfusion-dependent thalassemia.

* Statistically significant results.

**Table 5.** Multivariable cox regression model results of significant factors affecting survivals in thalassemia patients (method: forward likelihood ratio (LR)).

Variables	Adjusted hazard ratio (95% CI)	*p* Value
Transfusion type (TDT vs. NTDT)	3.68 (1.39–9.72)	0.008*
Mean serum ferritin level (≥3000 ng/mL vs. <3000 ng/mL)	4.18 (2.20–7.92)	<0.001*

LVEF, left ventricular ejection fraction; NTDT, non-transfusion-dependent thalassemia; TDT, transfusion-dependent thalassemia.

* Statistically significant results.

## Discussion

This study includes a diverse cohort of patients with thalassemia, including both alpha-thalassemia and beta-thalassemia, which are prevalent in Thailand and Southeast Asia. The analysis of survival factors revealed that TDT and iron overload are the predominant factors associated with diminished survival. Notably, infections and cardiac complications were the major contributors to mortality. These findings emphasize that clinical severity, rather than the specific type of thalassemia, plays a pivotal role in survival outcomes.

Cardiomyopathy is a well-documented complication of TDT, and a recognized factor associated with poorer survival in patients with TDT [7,9,14,26]. Transfusional iron overload is the main cause of cardiac hemochromatosis. An earlier study conducted in Thailand in 1987, when iron chelation therapy was not widely available, reported a high prevalence of cardiomyopathy, reaching 58% [26]. In line with these findings, our study identified a mean serum ferritin level ≥3000 ng/mL as a significant factor associated with poorer survival, aligning with previous investigations [8,13]. A study by Borgna-Pignatti et al. demonstrated that a serum ferritin level exceeding 2500 ng/mL was associated with reduced survival [8]. These results underscore the critical role of effective iron chelation in improving survivals.

Research from the United Kingdom has highlighted a marked improvement in survival among patients with thalassemia major, primarily attributable to a decline in deaths related to cardiac iron overload following the widespread availability of iron chelation therapy [11]. In our study, the prevalence of reduced LVEF was 3.4%, with 16.4% of patients having mean serum ferritin levels ≥3000 ng/mL. The prevalence of cardiomyopathy was lower when compared to a previous cross-sectional study conducted at our institute in 2011, which reported an 8% prevalence of cardiomyopathy [27]. Both of these figures were significantly lower than those reported in the aforementioned 1987 study [26]. The diverse study population of both TDT and NTDT may have contributed to these findings and could reflect the positive effects of widely available iron chelation therapy in reducing the risk of iron overload and cardiomyopathy.

Results from the univariable analysis indicated that significant factors associated with survival were the type of thalassemia, transfusion dependency status, mean pre-transfusion Hb levels and mean serum ferritin levels. In the subsequent multivariable analysis, both TDT and a mean serum ferritin level ≥3000 ng/mL were found to be independent factors associated with poorer survival. It is worth noting that the enhanced survival observed in patients with NTDT may have been influenced by the relatively high proportion of individuals with Hb H disease (301 out of 789 patients, 38.1%), who generally present with milder anaemia and a less severe disease spectrum. Additionally, it is crucial to acknowledge that the definition of NTDT can vary across different studies [21]. In this study, NTDT was defined as requiring less than an average of three transfusions per year, thus representing the milder end of the disease spectrum.

A recent study conducted on 537 patients with transfusion-dependent beta-thalassemia in Cyprus revealed that male gender and milder beta-globin gene (*HBB*) genotype were significantly associated with poorer outcomes [28]. The study suggested that delayed initiation of transfusion and management strategies may have contributed to the adverse long-term effects of insufficiently treated thalassemia, resulting in poorer survival rates. The identification of *HBB* genotype could assist in treatment planning and improve long-term outcomes [28]. However, in our study, thalassemia diagnosis for most patients was made by Hb analysis, precluding patient categorization based on genotype. Future research focusing on the survival, morbidity and mortality of patients with beta-thalassemia, particularly Hb E/beta-thalassemia, which presents with varying degrees of severity, is warranted. Such studies can provide valuable treatment recommendations for this patient population.

Infections were the leading cause of death in our study. The findings were different from several previous studies in which cardiomyopathy was the leading cause [7,9,14,26]. However, our findings align with a study conducted in South India, focusing on paediatric patients with thalassemia, where infections were identified as the primary cause of death [29]. Patients with thalassemia are inherently prone to infections, attributed to chronic blood transfusion, iron overload and splenectomy. Chronic blood transfusion can compromise immunity by transfusion-induced immunomodulation (TRIM) [30]. Moreover, transfusions contribute to iron-overload mediated toxicity in macrophages/monocytes, and dampen the inflammatory response to infections by increasing IL-10 production [31,32]. Splenectomy is also a recognized risk factor for serious infections [33], and it is associated with the development of pulmonary hypertension and thrombosis which is associated with reduced survival [34–36]. In our patient cohort, the high percentage of splenectomized patients in the deceased group (40 out of 65 patients, 61.5%) likely played a role in the heightened risk of severe infections. These findings underscore the importance of infection prevention, early detection, and prompt treatment, particularly in splenectomized patients, to improve survival in the thalassemia population in our region.

The strength of this study was the diverse cohort of thalassemia patients with different severity and coverage of both alpha-thalassemia and beta-thalassemia. However, it is imperative to acknowledge the study’s limitations, and certain results warrant cautious interpretation. Firstly, the Pediatrics Registry covered a shorter timeframe compared to the Adult Registry, and a higher proportion of younger patients presented with Hb H disease. This discrepancy may be attributed to older patients with Hb H disease receiving treatment at primary healthcare facilities due to the milder nature of their condition. Secondly, patients who succumbed early in the course of their illness and were not referred to our institution may not have been captured in the study, potentially leading to an erroneously longer survival estimate across all patient groups. Thirdly, the recording of complications was retrospective, potentially resulting in an underestimation of the true incidence of each complication.

In summary, this study underscores TDT and iron overload as the primary factors associated with poorer survival in patients with thalassemia. The major causes of death were infections and cardiac complications. The principal contributors to mortality were infections and cardiac complications. More extensive studies over a longer period with an emphasis on globin genotypes and treatment strategies will be instrumental in further substantiating the enhancements in survival achieved through optimal transfusion practices, effective iron chelation, novel treatments, prevention of infections and supportive care measures.

## Data Availability

The medical records of the patients used in this analysis contain identifiable human subject data, which cannot be disseminated under the terms of the IRB and data use agreements with contributing institutions. For analyses of patient-level identifiable data within our trusted research environment, please email the corresponding author.
